# Comparison between epidural and intravenous analgesia effects on disease-free survival after colorectal cancer surgery: a randomised multicentre controlled trial

**DOI:** 10.1016/j.bja.2021.04.002

**Published:** 2021-05-07

**Authors:** Wiebke Falk, Anders Magnuson, Christina Eintrei, Ragnar Henningsson, Pär Myrelid, Peter Matthiessen, Anil Gupta

**Affiliations:** 1 Department of Anaesthesiology and Intensive Care, School of Medical Sciences, Örebro University, Örebro, Sweden; 2 Clinical Epidemiology and Biostatistics, School of Medical Sciences, Örebro University, Örebro, Sweden; 3 Department of Anaesthesiology and Intensive Care, Linköping University, Linköping, Sweden; 4 Department of Anaesthesiology and Intensive Care, Central Hospital Karlstad, Karlstad, Sweden; 5 Department of Biomedical and Clinical Sciences, Linköping University, Linköping, Sweden; 6 Department of Surgery, Linköping University Hospital, Linköping, Sweden; 7 Department of Surgery, School of Medical Sciences, Örebro University, Örebro, Sweden; 8 Department of Physiology and Pharmacology, Karolinska Institutet and Karolinska University Hospital, Stockholm, Sweden

**Keywords:** colorectal cancer, disease-free survival, minimally invasive surgery, open surgery, patient-controlled intravenous analgesia, recurrence, thoracic epidural analgesia

## Abstract

**Background:**

Thoracic epidural analgesia (TEA) has been suggested to improve survival after curative surgery for colorectal cancer compared with systemic opioid analgesia. The evidence, exclusively based on retrospective studies, is contradictory.

**Methods:**

In this prospective, multicentre study, patients scheduled for elective colorectal cancer surgery between June 2011 and May 2017 were randomised to TEA or patient-controlled i.v. analgesia (PCA) with morphine. The primary endpoint was disease-free survival at 5 yr after surgery. Secondary outcomes were postoperative pain, complications, length of stay (LOS) at the hospital, and first return to intended oncologic therapy (RIOT).

**Results:**

We enrolled 221 (110 TEA and 111 PCA) patients in the study, and 180 (89 TEA and 91 PCA) were included in the primary outcome. Disease-free survival at 5 yr was 76% in the TEA group and 69% in the PCA group; unadjusted hazard ratio (HR): 1.31 (95% confidence interval [CI]: 0.74–2.32), *P*=0.35; adjusted HR: 1.19 (95% CI: 0.61–2.31), *P*=0.61. Patients in the TEA group had significantly better pain relief during the first 24 h, but not thereafter, in open and minimally invasive procedures. There were no differences in postoperative complications, LOS, or RIOT between the groups.

**Conclusions:**

There was no significant difference between the TEA and PCA groups in disease-free survival at 5 yr in patients undergoing surgery for colorectal cancer. Other than a reduction in postoperative pain during the first 24 h after surgery, no other differences were found between TEA compared with i.v. PCA with morphine.

**Editor's key points**

• Retrospective studies suggest that thoracic epidural analgesia improves survival after curative surgery for colorectal cancer compared with systemic opioid analgesia.
• In this prospective multicentre study, patients undergoing colorectal cancer surgery were randomised to thoracic epidural analgesia or patient-controlled i.v. analgesia with morphine.
• There was no significant difference in the primary endpoint of disease-free survival at 5 yr after surgery despite better early analgesia in the epidural group.
• Future studies of the effects of anaesthetic technique on oncological outcomes should focus on other anaesthetic interventions, such as total intravenous anaesthesia with propofol compared with volatile anaesthesia.

Colorectal cancer is the third most common cancer worldwide, estimated to have caused ∼1 000 000 deaths in 2020.^1^ Surgery is the mainstay of treatment for solid tumours, such as colorectal cancer. In the past decade, minimally invasive techniques have become routine procedures and perioperative care has improved after the introduction of enhanced recovery after surgery (ERAS) programmes.^2^^,^^3^ Current guidelines recommend thoracic epidural analgesia (TEA) for management of postoperative pain after open colorectal surgery.^4^ However, TEA has not been shown convincingly to reduce postoperative morbidity or mortality,^5^ and the use of TEA is no longer recommended for minimally invasive surgery procedures in current guidelines.^4^

Prevention of postoperative complications after colorectal surgery, specifically in patients requiring adjuvant chemotherapy, is important, as delayed chemotherapy decreases survival.^6^ Therefore, return to intended oncologic therapy (RIOT) might be a useful endpoint investigating analgesic methods.^7^^,^^8^ Evidence that TEA might prolong overall and disease-free survival after surgery for colorectal surgery remains unclear and contradictory, and is based solely on retrospective studies,^9^, ^10^, ^11^ as there are no prospective RCTs investigating survival after colorectal cancer surgery with or without TEA to our knowledge.

The primary aim of this prospective, randomised, multicentre study was to assess whether TEA improves disease-free survival at 5 yr compared with patient-controlled analgesia (PCA) with morphine after elective open or minimally invasive surgery for colorectal cancer in an established ERAS programme. The secondary aims were to evaluate short-term postoperative outcomes, including pain, complications, length of hospital stay (LOS), and RIOT.

## Methods

### Ethical approval

The Regional Ethics Committee in Linköping, Sweden approved the study on January 26, 2011 (registration number: 2010/415-31). It was registered in an international directory, www.clinicaltrials.gov (identifier NCT01318161). Informed verbal and written consent were obtained from all patients before randomisation.

### Randomisation and blinding

Patients with suspected colorectal cancer were randomly allocated to TEA or PCA in a 1:1 ratio using concealed allocation, and stratified by study centre, type of cancer (colon/rectum), and type of surgery (open/minimally invasive) to ensure homogeneity between groups. Randomisation was performed centrally from Linköping using computer-generated numbers inserted into opaque, sealed envelopes. Neither the patient, surgeon, anaesthesiologist, nor the attending nurse was blinded to the method of analgesia because of medical, ethical, and logistical reasons.

### Patients and settings

We planned to include 300 patients from three hospitals in Central Sweden (Linköping University Hospital, Örebro University Hospital, and Karlstad Central Hospital). Patients aged 30–80 yr, ASA physical status 1–3, and scheduled for elective open or minimally invasive curative colorectal cancer surgery were eligible for inclusion. Exclusion criteria were known metastasis or malignant recurrence, acute surgery, chronic opioid or corticosteroid medication, known immunological disease, or contraindication to epidural or i.v. morphine analgesia. Patients judged to have malfunctioning TEA during or after surgery were excluded *a priori* from data analysis.

### Surgery

The surgical technique, open or minimally invasive, was chosen according to patient and tumour characteristics. Open surgery included laparotomy through a midline abdominal incision. Minimally invasive surgery included a small suprapubic Pfannenstiel or small midline incision to deliver the surgical specimen, and included both laparoscopic and robot-assisted laparoscopic multiport techniques. All patients were cared for according to the principles of ERAS,^4^ which is a routine at the participating hospitals. When minimally invasive surgery could not be completed because of tumour characteristics or surgical difficulty, the operative procedure was converted to open *via* a laparotomy.

### Anaesthesia

General anaesthesia was induced with fentanyl (2–3 μg kg^−1^) and propofol (2–3 mg kg^−1^). Rocuronium (0.6 mg kg^−1^) was used as a neuromuscular blocking drug for intubation. Maintenance of anaesthesia was accomplished using sevoflurane in oxygen/air (*F*io_2_ 0.3–0.5), and intermittent controlled ventilation was used in all patients.

Before induction of general anaesthesia, patients in the TEA group received an epidural catheter, which was inserted at the thoracic 10–12 interspace and tested for correct placement. Perioperative epidural analgesia was achieved according to local hospital routines using a combination of local anaesthetic and opioid.

Patients in the PCA group received bolus doses of fentanyl 25–50 μg for intraoperative analgesia at the discretion of the assigned anaesthesiologist or nurse anaesthetist. Morphine i.v. was given at the end of surgery for management of early postoperative pain.

### Postoperative analgesia

Patients in the TEA group were excluded from the study if analgesia was judged to be inadequate (>10 mg morphine i.v. required immediately after surgery) in the postoperative ward. All patients received paracetamol 1 g i.v. every 6 h. Patients in the TEA group received an infusion of local anaesthetic and opioid according to local hospital routines. In the PCA group, patients received morphine 1 mg on demand with a lockout period of 6 min up to a maximum dose of 10 mg h^−1^. These analgesic regimes were applied for up to 72 h postoperatively. NSAIDs were administered if needed for pain management. Oral opioids were given to all patients if needed after discontinuation of TEA or PCA analgesia.

### Postoperative follow-up

Pain intensity was assessed by use of a numeric rating scale (NRS) (score 0–10, where 0=no pain and 10=worst imaginable pain) twice daily (morning and late afternoon) starting on the day after surgery (Day 1). Dedicated research nurses assessed postoperative recovery (adequate mobilisation in relation to preoperative status, tolerance of oral feeding, and pain control by oral analgesics) at Days 3–5 and complications (cardiovascular, respiratory, infectious, surgical, and urological) for 30 days after surgery. Patients were monitored with annual CT scans for tumour recurrence or metastasis. When occurring, the date of death was recorded.

### Study endpoints

The primary endpoint was disease-free survival at 5 yr, consistent with the Standardised Endpoints in Perioperative Medicine (StEP) trials in the context of onco-anaesthesia.^12^ The primary endpoint as originally registered was 5-yr all-cause mortality (NCT01318161); we analysed and reported data as disease-free survival to align with the StEP recommendations. Patients were censored at the date of the last visit if no event (recurrence or death) occurred. Secondary endpoints were intensity of postoperative pain measured using NRS, and rescue analgesia, recovery, and complications within 30 days, LOS, and time to RIOT.

### Statistical analysis

A power calculation was performed before the start of the study. Assuming event risk in the non-epidural group to be 40% at 5 yr, we were interested in a reduction by 15% as clinically relevant in the epidural group. Assuming α=0.05 and β=80%, we determined that 300 subjects would be needed. All opioid analgesics given perioperatively were converted into oral morphine equivalents (OMEs) to allow comparison.^13^

Continuous variables were analysed by *t*-test, or by Mann–Whitney *U*-test if not normally distributed. Binary variables were analysed by χ^2^ or Fisher's exact test, as appropriate.

Kaplan–Meier and unadjusted and adjusted Cox regression were used to visualise and compare disease-free survival between the TEA and PCA groups. Cox regression was adjusted for age, sex, BMI, ASA physical status, study centre (Linköping, Örebro, or Karlstad), type of cancer (rectal or colon), type of surgery (open, minimally invasive, or converted), tumour T and N stages, and neo-adjuvant and adjuvant treatments. All variables were analysed on categorical scale; age was categorised into <65, 65 to <75, and ≥75 yr; and BMI as WHO classification <25, 25 to <30, and ≥30 kg m^−2^. Because of the sparse number of subjects (and outcomes), T stage T0, T1, and T2 were collapsed to one category; adjustment was also performed with a backward stepwise Cox regression at significance level 0.20 for selecting variables. Proportional hazard assumption was tested by phtest^14^ in STATA (StataCorp, College Station, TX, USA) using the Schoenfeld residuals, and if violated, stratified Cox regression was used. The association measure was hazard ratio (HR) with 95% confidence intervals (CIs). Sensitivity analyses were performed to be able to analyse as intention-to-treat principle, including patients not followed up because of absence of pathology report confirming colorectal cancer or patients lost to follow-up. Four sensitivity analyses were done with Cox regression: (i) setting missing to having an event at 1 yr, (ii) having an event at 2.5 yr, (iii) having an event at 4 yr, and (iv) having no event.

A random-intercept linear mixed model with first-order autoregressive correlation structure was used to evaluate postoperative pain scores. Fixed factors were study group (TEA or PCA), time of pain registration, and their interaction. Subgroup analyses were conducted amongst patients subjected to open or minimally invasive surgery, with and without those converted from minimally invasive surgery to open. Because of some violation of the normal assumption, analyses were also conducted after log_10_ transformation as sensitivity analysis. Statistical significance was considered at a *P*-value <0.05. Data were analysed using IBM SPSS statistics, version 25 (IBM Corp., Armonk, NY, USA) and STATA release 14 (Stata Corp).

## Results

**Fig 1:**
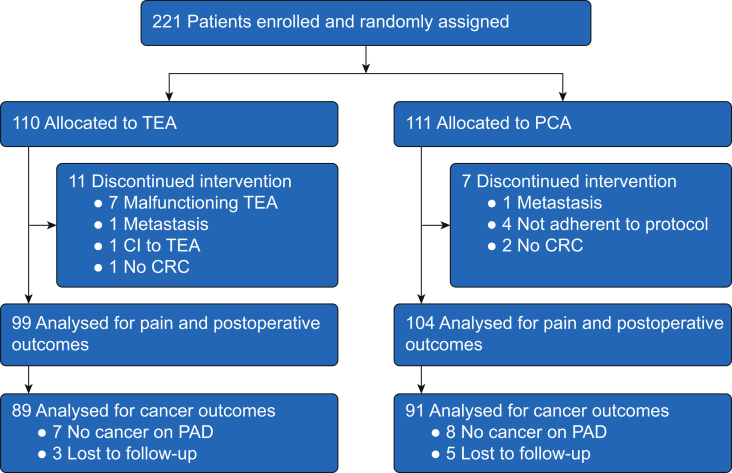
Consolidated Standards of Reporting Trials diagram. CI, contraindication; CRC, colorectal cancer; PAD, pathological anatomical diagnosis; PCA, patient-controlled i.v. analgesia; TEA, thoracic epidural analgesia.

During the period from June 2011 to May 2017, a total of 221 patients were recruited; 110 patients were randomised to TEA and 111 patients to PCA. Of these, 18 patients were excluded, leaving a total of 203 subjects, 99 subjects in group TEA and 104 subjects in group PCA (Fig 1). For the primary outcome, a further 15 subjects were excluded because the pathology report did not confirm invasive colorectal cancer, and eight subjects were lost to follow-up (Fig 1). The study was terminated prematurely in mid-2017 because of difficulty in recruitment during the study period, specifically in the past 2 yr with predominance for minimally invasive surgery and an increasing reluctance of colleagues to use TEA in these patients. Subject characteristics, duration of surgery, incidence of preoperative radiation therapy, and tumour/node/metastases stage were similar between groups (Table 1).

**Table 1:** Patient and surgery characteristics

	TEA (*n*=99)	PCA (*n*=104)
Age (yr), mean (range)	67.9 (41–80)	67.2 (39–81)
Women^†^	31 (31)	44 (42)
BMI (kg m^−2^)∗	27.0 (4.0)	26.3 (4.6)
<25, normal^†^	31 (31)	45 (43)
25 to <30, pre-obesity^†^	49 (49)	43 (41)
≥30, obesity^†^	19 (19)	16 (15)
ASA physical status^†^		(*n*=103)
1	31 (31)	28 (27)
2	52 (53)	66 (64)
3	16 (16)	9 (9)
Study centre^†^		
Linköping	46 (46)	51 (49)
Örebro	46 (46)	48 (46)
Karlstad	7 (7)	5 (5)
Type of cancer^†^		
Rectal	49 (49)	54 (52)
Colon	50 (51)	50 (48)
Type of surgery^†^		
Open	60 (61)	60 (58)
Minimally invasive	34 (34)	34 (33)
Converted	5 (5)	10 (10)
T stage^†^		(*n*=103)
No cancer	7 (7)	8 (8)
ypT0	2 (2)	2 (2)
T1	6 (6)	6 (6)
T2	18 (18)	14 (14)
T3	58 (59)	60 (58)
T4	8 (8)	13 (13)
N stage^†^		
No cancer	7 (7)	8 (8)
N0	59 (60)	49 (47)
N1	19 (19)	36 (35)
N2	14 (14)	11 (11)
Preoperative radiotherapy (only rectal cancer)^†^	33 (33)	34 (34)
Adjuvant treatment^†^	(*n*=98) 37 (38)	(*n*=102) 46 (45)
Duration of surgery (min), median (IQR)	220 (150–281)	197 (152–276)
Comorbidities^†^		
Hypertension	45 (46)	44 (42)
IHD	6 (6)	8 (8)
Cardiac failure	1 (1)	3 (3)
Diabetes mellitus	19 (19)	13 (13)
CKD	5 (5)	3 (3)
COPD	3 (3)	6 (6)

Values denote ∗mean (standard deviation) or ^†^*n* (%) of patients unless otherwise stated. Continuous variables were analysed by *t*-test if normally distributed, or by Mann–Whitney *U*-test if not normally distributed. Categorical variables were analysed by χ^2^ or Fisher's exact test when appropriate.

CKD, chronic kidney disease; COPD, chronic obstructive pulmonary disease; IHD, ischaemic heart disease; IQR, inter-quartile range; PCA, patient-controlled opioid analgesia; TEA, thoracic epidural analgesia.

### Primary outcome

**Fig 2:**
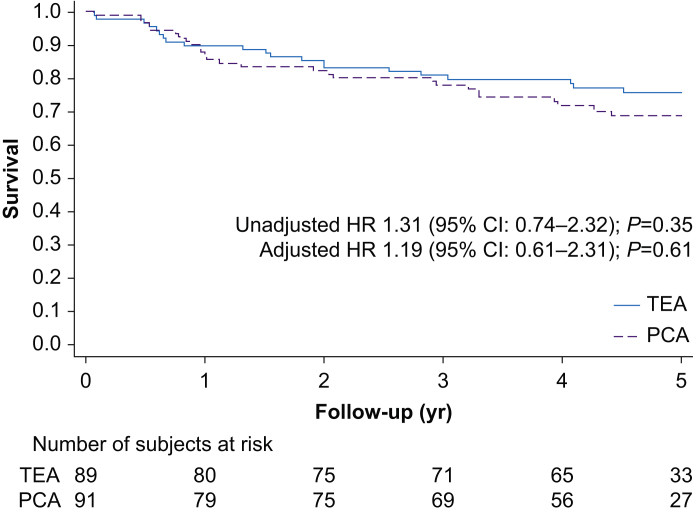
Kaplan–Meier plots for patients undergoing surgery for colorectal cancer with thoracic epidural analgesia (TEA; solid line) or with patient-controlled analgesia with morphine (PCA; dashed line). CI, confidence interval; HR, hazard ratio.

A total of 48 events were recorded in 180 subjects (26.7%) during the 5 yr follow-up. Disease-free survival at 5 yr in group TEA was 76% compared with 69% in group PCA (*P*=0.35). In the TEA group (*n*=89), we recorded 21 first events, 18 subjects had local or metastatic recurrence, and nine subjects died, three without confirmed recurrence before death. In the PCA group (*n*=91), there were 27 first events, with 25 cases of local or metastatic recurrence and 14 deaths, two deaths without known recurrence. Kaplan–Meier curves for disease-free survival in the TEA and PCA groups are presented in Fig 2. The median follow-up time was 4.9 (inter-quartile range [IQR]: 3.0–5.0) yr, and 88% (116/132) of non-events subjects had 4 yr or longer follow-up. Cox regression showed unadjusted HR 1.31 (95% CI: 0.74–2.32), *P*=0.35; adjusted HR 1.19 (95% CI: 0.61–2.31), *P*=0.61; and stepwise adjusted HR 1.15 (95% CI: 0.61–2.15), *P*=0.66 comparing the PCA and TEA groups (Table 2). The four sensitivity analyses, including 203 subjects (99 TEA and 104 PCA) showed similar or somewhat lower HR, ranging from HR 1.09 (95% CI: 0.56–2.12), *P*=0.79 to HR 1.14 (95% CI: 0.68–1.92), *P*=0.62 (Supplementary Tables S1 and S2).

**Table 2:** Cox regression for disease-free survival, 48 events amongst 180 patients

	*N*	Outcome		Unadjusted (*n*=180)		Adjusted (*n*=176)		Stepwise† (*n*=176)	
		*n* (%)	Rate∗	HR (95% CI)	*P*-value	HR (95% CI)	*P*-value	HR (95% CI)	P-value
TEA	89	21 (23.6)	5.9	Reference		Reference		Reference	
PCA	91	27 (29.7)	7.8	1.31 (0.74–2.32)	0.35	1.19 (0.61–2.31)	0.61	1.15 (0.61–2.15)	0.66
Age (yr)									
<65	63	18 (28.6)	7.4	Reference		Reference		Reference	
65 to <75	80	14 (17.5)	4.3	0.59 (0.29–1.16)	0.14	0.70 (0.32–1.50)	0.36	0.80 (0.38–1.64)	0.54
≥75	37	16 (43.2)	12.5	1.67 (0.85–3.28)	0.13	2.30 (1.03–5.16)	0.042	2.14 (1.07–4.27)	0.032
Sex									
Women	65	15 (23.1)	5.6	Reference		Reference		Reference	
Men	115	33 (28.7)	7.6	1.36 (0.74–2.50)	0.29	2.04 (1.00–4.14)	0.049	1.68 (0.87–3.22)	0.12
Study centre									
Örebro	90	16 (17.8)	4.4	Reference		Reference		Reference	
Linköping	80	30 (37.5)	10.1	2.27 (1.24–4.17)	0.008	1.62 (0.80–3.29)	0.18	1.70 (0.91–3.17)	0.096
Karlstad	10	2 (20.0)	4.5	1.02 (0.23–4.45)	0.98	0.49 (0.10–2.37)	0.38	0.65 (0.14–2.96)	0.58
Type of cancer									
Rectal	98	26 (26.5)	6.6	Reference		Reference			
Colon	82	22 (26.8)	7.1	1.05 (0.60–1.86)	0.86	1.53 (0.60–3.85)	0.37		
Type of surgery									
Laparoscopic	76	13 (17.1)	4.2	Reference		Reference			
Open/converted	104	35 (33.6)	8.9	2.07 (1.09–3.91)	0.025	1.23 (0.59–2.56)	0.58		
BMI (kg m^−2^)									
<25 normal	67	20 (29.8)	7.6	Reference		Reference			
25 to <30 pre-obesity	81	20 (24.7)	6.3	0.83 (0.45–1.55)	0.57	0.84 (0.42–1.71)	0.64		
≥30 obesity	32	8 (25.0)	6.7	0.89 (0.39–2.03)	0.78	1.18 (0.46–3.01)	0.73		
ASA physical status	(*n*=179)								
1	53	13 (24.5)	6.1	Reference		Reference			
2	107	30 (28.0)	7.3	1.17 (0.61–2.24)	0.64	0.78 (0.37–1.65)	0.52		
3	19	5 (26.3)	6.8	1.13 (0.40–3.18)	0.81	0.82 (0.23–2.88)	0.75		
T stage	(*n*=179)								
T0/T1//T2	45	3 (6.7)	1.5	Reference		Reference		Reference	
T3	113	33 (29.2)	7.5	5.00 (1.53–16.3)	0.008	3.00 (0.84–10.8)	0.091	3.57 (1.06–12.0)	0.040
T4	21	12 (57.1)	22.6	13.7 (3.85–48.7)	<0.001	5.84 (1.23–27.6)	0.026	7.51 (2.01–28.0)	0.003
N stage									
N0	105	15 (14.3)	3.4	Reference		Reference		Reference	
N1	52	22 (42.3)	12.1	3.45 (1.79–6.66)	<0.001	1.90 (0.81–4.44)	0.14	2.48 (1.22–5.03)	0.012
N2	23	11 (47.8)	15.2	4.26 (1.95–9.28)	<0.001	2.21 (0.89–5.50)	0.088	3.13 (1.38–7.08)	0.006
Preoperative radiotherapy	66	21 (31.8)	8.2	1.34 (0.76–2.38)	0.31	1.76 (0.73–4.25)	0.21		
Adjuvant treatment	(*n*=178) 79	33 (41.8)	11.9	3.12 (1.70–7.76)	<0.001	1.73 (0.67–4.49)	0.26		

∗ Rates, number of events per 100 person-years.

† Backward stepwise regression with significance level of 0.20.

### Secondary outcomes

**Fig 3:**
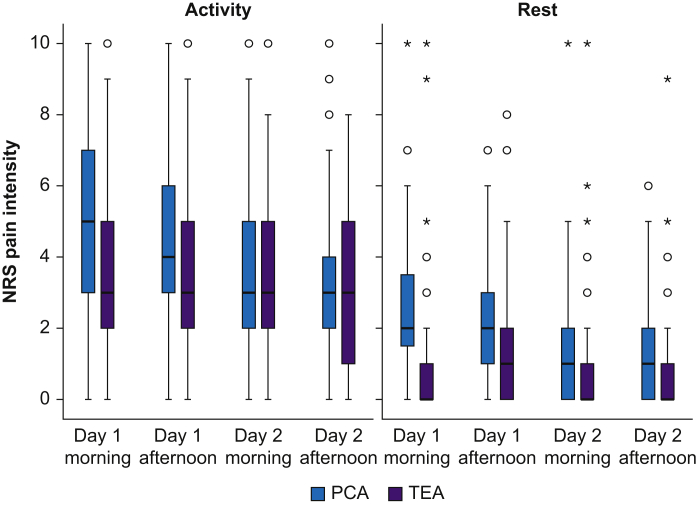
Box and whisker plot for pain intensity at rest and activity measured by numeric rating scale (NRS; 0=no pain and 10=worst imaginable pain) in the first 2 postoperative days. *P*<0.05 on Day 1 between groups, at rest and on activity. PCA, patient-controlled analgesia; TEA, thoracic epidural analgesia.

Significantly lower pain intensity on activity was seen in the TEA group in the morning of Postoperative day (POD) 1 compared with PCA with morphine (mean difference: –1.8; 95% CI: –2.4 to –1.1; *P*<0.001) after both open (mean difference: –1.4; 95% CI: –2.3 to –0.5; *P*=0.002) and minimally invasive (mean difference: –2.6; 95% CI: –3.7 to –1.5; *P*<0.001) surgery (Supplementary Table S3). There was no statistically significant difference in NRS pain scores at activity between groups on POD 2. Pain intensity at rest was also significantly lower after both open (mean difference: –1.4; 95% CI: –2.0 to –0.7; *P*<0.001) and minimally invasive (mean difference: –2.2; 95% CI: –2.9 to –1.5; *P*<0.001) surgery with TEA compared with PCA on POD 1 and for open surgery on POD 2 (mean difference: –0.8; 95% CI: –1.5 to –0.1; *P*=0.018) (Supplementary Table S4). Pain intensity with activity and rest in both groups is presented as a box plot in Fig 3. Subjects in the TEA group received significantly less OMEs as rescue analgesia in the postoperative ward (Table 3).

**Table 3:** Perioperative data and postoperative complications

	*n*	TEA (*n*=99)	*n*	PCA (*n*=104)	*P*-value
OME during surgery (mg)	83	35 (30–50)	103	101 (78–130)	<0.001
OME in PACU (mg)	98	0 (0–0)	104	9.5 (0–29.25)	<0.001
Vasoactive drugs (during surgery)	99	87 (87.9)	104	68 (65.4)	<0.001
Fluids during surgery (ml), mean (sd)	97	2306 (1259)	104	2041 (1113)	0.11
Anti-emetics					
POD 0	98	33 (34)	104	39 (37)	0.57
POD 1	98	20 (19)	104	29 (28)	0.16
POD 2	97	21 (22)	103	26 (25)	0.55
POD 3–5					
Adequately mobilised	97	92 (95)	104	97 (93)	0.65
Adequate PO intake	96	79 (82)	103	78 (76)	0.27
Pain controlled orally	95	88 (93)	104	97 (93)	0.86
Postoperative complications					
Any complication	97	39 (40)	104	40 (38)	0.76
Anastomotic leakage	97	10 (10)	104	7 (7)	0.35
Intestinal paralysis	97	15 (16)	104	11 (11)	0.30
Wound infection	97	14 (14)	104	10 (10)	0.29
Pneumonia	97	1 (1)	104	1 (1)	0.96
Problems in micturition	97	9 (9)	104	7 (7)	0.51
LOS (days)					
Overall	99	6 (5–10)	103	6 (5–8)	0.34
Colon	50	5 (4–8)	49	6.5 (5–9.75)	0.97
Colon open	33	5 (4–8.5)	31	6 (4–7)	0.98
Colon MIS	14	4.5 (3–6.5)	13	4 (3–6.5)	0.98
Rectum	49	7 (6–14.5)	54	6.5 (5–9.75)	0.11
Rectum open	27	10 (6–15)	27	7 (5–8)	0.04
Rectum MIS	20	6 (5–8.75)	21	6 (5–8.5)	0.57
RIOT (days)	37	44 (33–54.5)	46	48 (36.5–57)	0.24
RIOT <8 weeks	37	30 (81)	46	34 (74)	0.44

Values denote median values (inter-quartile range) or *n* (%) of patients if nothing else was stated. Continuous variables were analysed by *t*-test if normally distributed, or by Mann–Whitney *U*-test if not normally distributed. Categorical variables were analysed by χ^2^ or Fisher's exact test when appropriate. Surgery converted from MIS to OS is not included in analysis of LOS for OS or MIS procedures. LOS, length of stay; MIS, minimal invasive surgery; OME, oral morphine equivalent; OS, open surgery; PO, per os; POD, postoperative day; RIOT, return to intended oncologic therapy; sd, standard deviation.

More subjects in the TEA group needed vasoactive drugs for haemodynamic stability during surgery compared with subjects in the PCA group (87.8% *vs* 65.4%; *P*<0.001). There were no differences in fluids administered during surgery or need for anti-emetic therapy postoperatively between groups. Recovery, defined by adequate mobilisation, tolerance of oral feeding, and pain control by oral analgesics on POD 3–5, was also similar between groups (Table 3).

The incidence of postoperative complications and LOS for all included subjects subdivided into type of cancer and type of surgery are shown in Table 3. Length of hospital stay was slightly longer for subjects receiving TEA for open rectal surgery, but no other differences were seen between groups.

In group TEA, 37 subjects (41.6%) were eligible for adjuvant oncologic therapy, whilst 46 subjects (50.6%) were eligible in group PCA. Time to RIOT was slightly shorter (median [IQR] 44 [33–54.5] *vs* 48 [36.5–57] days), and a greater proportion of subjects could start adjuvant chemotherapy within 8 weeks postoperatively (82% *vs* 74%) in group TEA compared with group PCA (Table 3); however, these differences did not reach statistical significance.

## Discussion

In this randomised study in patients undergoing colorectal cancer surgery, we found no significant effect of TEA compared with PCA with morphine on disease-free survival at 5 yr. Pain intensity with activity during the first 24 h was lower in the TEA group in both open and minimally invasive surgeries, but not thereafter. No differences were found in any other recorded parameters.

Several mechanisms have been proposed for better long-term outcomes of epidural compared with i.v. opioid analgesia in cancer surgery.^15^ Firstly, TEA reduces the need for systemic opioids for pain management. Opioids have been shown to promote angiogenesis,^16^ to increase tumour cell proliferation,^17^ and to suppress natural killer cells, a primary defence against circulating cancer cells.^18^ Secondly, surgical trauma and subsequent perioperative pain are known to trigger a stress response, which leads to inflammation and immunosuppression.^19^ Thoracic epidural analgesia has been shown to reduce this stress response during major surgery and may promote earlier recovery, hospital discharge, and earlier start of adjuvant chemotherapy. These beneficial effects of TEA have been thought to improve long-term survival,^20^ and this hypothesis has been tested in several studies in patients undergoing colorectal cancer surgery. All published studies have been retrospective, or a secondary analysis of data from prospective trials with a non-cancer primary endpoint, and the results are conflicting.^9^^,^^10^^,^^21^^,^^22^ The primary objective of our study was to determine cancer recurrence, metastases, or death after colorectal cancer surgery in patients randomised to TEA or PCA. We found no significant difference in disease-free survival between groups during the 5 yr follow-up. However, the current model is very unlikely to be able to prove a difference, and an adequately powered study might be difficult to perform because of an increasing volume of minimally invasive surgery, where TEA is not recommended today. Based on our results, with a difference in disease-free survival of ∼7% between the TEA and PCA groups, a *post hoc* power analysis reveals that 635 subjects per group need to be recruited to demonstrate a difference with 80% power and a significance level of 5%. One large trial in patients undergoing breast cancer surgery has been performed and found no benefit of paravertebral block.^23^

Recently, RIOT has been discussed as an important landmark for prediction of long-term outcome after ovarian cancer surgery^7^ and colorectal cancer surgery.^6^ In the present study, we did not find a significant difference in the time to RIOT or the percentage of patients who could return to adjuvant oncologic therapy within 8 weeks after surgery. However, these results were based on analysis of only a small number of patients receiving adjuvant chemotherapy. The effect of TEA on time to RIOT after colorectal cancer surgery should be investigated in future trials with adequate power. Such a study has been registered in an international database and is expected to start soon (NCT04493905).

Thoracic epidural analgesia has been shown to reduce pain intensity compared with PCA morphine after abdominal surgery.^24^ Our findings were similar. However, we found that the mean difference in pain intensity between groups was small, both at rest and on activity in those having open surgery and minimally invasive surgery. The benefit of TEA was limited to the first 24 h after surgery. Superior pain relief early after surgery in the TEA group did not lead to a significant reduction in postoperative complications or LOS. Older studies have reported faster recovery and reduced complications in patients having TEA compared with systemic analgesia.^20^ Thoracic epidural analgesia, in the context of an established ERAS pathway, might not offer any significant benefit today, as other measures may overweigh the known advantages of TEA. There is good evidence that ERAS programmes significantly reduce postoperative morbidity, and consequently LOS compared with traditional care.^25^^,^^26^ Multimodal analgesia is a crucial part of the ERAS concept. However, TEA is resource demanding, there is a risk for failure, and it may rarely result in serious complications. Therefore, alternative methods should be considered and re-evaluated.^27^

### Study limitations

This was a pragmatic trial performed in three centres in Sweden. Thoracic epidural analgesia was provided according to the hospital routines, not specifically modified to a study protocol, thus improving the generalisability of the trial findings. Patients were cared for according to the ERAS principles that are routine in the participating hospitals. However, the surgical technique changed during the enrolment period. In 2011, the majority of patients underwent open surgery, whilst minimally invasive surgery had become standard in 2017. Although long-term outcomes after open surgery and minimally invasive surgery have been shown to be equivalent in previous studies,^28^^,^^29^ we decided to stratify for the surgical technique in the randomisation process. We included both colon and rectal cancers, and there was an even distribution of these cancers between the study groups. However, we did not consider the exact cancer phenotype and cannot exclude that its skewed distribution might have influenced the results, as only a small number of subjects were included in this study. Future trials should account for different phenotypes and also differentiate between colon and rectal cancers because of major differences in the anatomy, complexity of surgery, and use of neo-adjuvant and adjuvant therapies,^30^ and also because a previous study suggested better outcomes for TEA after rectal but not colon surgery.^10^

When this study was designed in 2010, we based the sample size calculation on an event rate of 40% at 5 yr in the control group and aimed to detect an absolute reduction of events by 15% based on mortality reduction of 9% in patients with rectal cancer and TEA.^10^ We made an assumption that exclusion of patients with failed epidurals may lead to a more pronounced effect of TEA on cancer outcome. However, excluding failed epidurals resulted in a deviation from the intention-to-treat principle that could be questioned. A further limitation is that the trial had to be stopped before all 300 planned patients had been included because of difficulties in recruiting patients. In the past years of enrolment, the majority of patients underwent minimally invasive surgery, and randomisation between TEA and PCA became increasingly difficult, as both surgeons and anaesthesiologists were reluctant to use TEA with minimally invasive surgery. With <200 patients included in the final analysis, the power of the study to detect a difference of 15% was just over 0.5, and therefore, the study is underpowered to answer the original question. Finally, there was a loss of patients after initial recruitment attributable to a non-cancer diagnosis (Fig 1). Therefore, we performed four sensitivity analyses. The HR remained similar or somewhat lower, indicating that patient dropouts did not alter the results significantly.

### Conclusions

Taking into account the aforementioned limitations, TEA for colorectal cancer surgery did not significantly impact disease-free survival at 5 yr. The only benefit we found of TEA over PCA with morphine was better pain relief during the first 24 h postoperatively, but not thereafter. Future research should focus on the effect of other anaesthetic interventions, such as TIVA with propofol compared with volatile anaesthesia that might affect oncological outcome.

## Authors' contributions

Study design: CE, AG

Data collection: WF, RH, PMy

Data analysis: WF, AM

Data interpretation: WF, AM, PMy, PMa, AG

Drafting of paper: WF, AM, RH, PMa, PMy, AG

Critical revision of paper: AG

Final approval of paper: all authors.
