# Supplementary material for: Comparison between epidural and intravenous analgesia effects on disease-free survival after colorectal cancer surgery: a randomised multicentre controlled trial
Source: Br J Anaesth. 2021 May 7;127(1):65–74. doi: 10.1016/j.bja.2021.04.002 (PMC8258969; doi:10.1016/j.bja.2021.04.002)
Supplement: Multimedia component 1 [file mmc1.docx]

**Table 1s. Sensitivity analyses with Cox regression for time to composite outcome (recurrence/ death) and five years follow up, among 203 patients.**

|  |  | Outcome | | Unadjusted  (n=203) | | Adjusted^2^  (n=198) | |
| --- | --- | --- | --- | --- | --- | --- | --- |
|  | N | n (%) | Rate^1^ | HR (95% CI) | P  value | HR (95% CI) | P  value |
| Sensitivity analyses 1, missing 23 subjects sets to event at 1 year | | | | | | | |
| TEA | 99 | 31 (31.3) | 8.5 | Reference |  | Reference |  |
| PCA | 104 | 40 (38.5) | 11.2 | 1.29 (0.80-2.06) | 0.29 | 1.14 (0.68-1.92) | 0.62 |
| Sensitivity analyses 2, missing 23 subjects sets to event at 2.5 year | | | | | | | |
| TEA | 99 | 31 (31.3) | 8.1 | Reference |  | Reference |  |
| PCA | 104 | 40 (38.5) | 10.6 | 1.28 (0.80-2.04) | 0.30 | 1.12 (0.66-1.88) | 0.67 |
| Sensitivity analyses 3, missing 23 subjects sets to event at 4 year | | | | | | | |
| TEA | 99 | 31 (31.3) | 7.8 | Reference |  | Reference |  |
| PCA | 104 | 40 (38.5) | 10.1 | 1.32 (0.82-2.10) | 0.25 | 1.12 (0.67-1.89) | 0.66 |
| Sensitivity analyses 4, missing 23 subjects sets to no event | | | | | | | |
| TEA | 99 | 21 (21.2) | 5.2 | Reference |  | Reference |  |
| PCA | 104 | 27 (26.0) | 6.6 | 1.26 (0.71-2.23) | 0.42 | 1.09 (0.56-2.12) | 0.79 |

^1^Rates=number of events per 100 person-years. ^2^Cox regression stratified on T and N stage and adjusted for Age (<65, 65 to <75, ≥75), sex, study centre, type of cancer, type of surgery, BMI (<25, 25 to <30, ≥30), ASA (I, II, III/IV), T stage (T0/TI/T2, /T3, T4), N stage (N0, N1, N2), neo-adjuvant therapy and adjuvant therapy

**Table 2s. Sensitivity analyses 2, Stratified Cox regression for time to composite outcome (recurrence/ death) and five years follow up, 71 (48+23) events among 203 patients.**

|  |  | Outcome | | Unadjusted  (n=203) | | Adjusted^2^  (n=198) | |
| --- | --- | --- | --- | --- | --- | --- | --- |
|  | N | n (%) | Rate^1^ | HR (95% CI) | P  value | HR (95% CI) | P  value |
|  |  |  |  |  |  |  |  |
| TEA | 99 | 31 (31.3) | 8.1 | Reference |  | Reference |  |
| PCA | 104 | 40 (38.5) | 10.6 | 1.28 (0.80-2.04) | 0.30 | 1.12 (0.66-1.88) | 0.67 |
| Age |  |  |  |  |  |  |  |
| <65 | 70 | 25 (35.7) | 9.6 | Reference |  | Reference |  |
| 65 to <75 | 90 | 24 (26.7) | 6.8 | 0.72 (0.41-1.26) | 0.24 | 0.78 (0.41-1.48) | 0.45 |
| ≥75 | 43 | 22 (51.2) | 15.4 | 1.61 (0.91-2.86) | 0.10 | 1.78 (0.87-3.66) | 0.12 |
| Sex |  |  |  |  |  |  |  |
| Women | 75 | 25 (33.3) | 8.6 | Reference |  | Reference |  |
| Men | 128 | 46 (35.9) | 9.9 | 1.15 (0.71-1.87) | 0.57 | 1.20 (0.68-2.12) | 0.52 |
| Study centre |  |  |  |  |  |  |  |
| Örebro | 94 | 20 (21.3) | 5.4 | Reference |  | Reference |  |
| Linköping | 97 | 47 (48.4) | 13.9 | 2.48 (1.47-4.19) | 0.001 | 1.47 (0.78-2.80) | 0.23 |
| Karlstad | 12 | 4 (33.3) | 8.0 | 1.48 (0.50-4.32) | 0.48 | 0.64 (0.19-2.14) | 0.47 |
| Type of cancer |  |  |  |  |  |  |  |
| Rectal | 103 | 31 (30.1) | 7.7 | Reference |  | Reference |  |
| Colon | 100 | 40 (40.0) | 11.3 | 1.40 (0.87-2.23) | 0.16 | 1.34 (0.62-2.91) | 0.46 |
| Type of surgery |  |  |  |  |  |  |  |
| Laparoscopic | 83 | 20 (24.1) | 6.2 | Reference |  | Reference |  |
| Open/converted | 120 | 51 (42.5) | 11.7 | 1.90 (1.13-3.19) | 0.015 | 1.09 (0.59-2.00) | 0.78 |
| Body mass index |  |  |  |  |  |  |  |
| <25 normal | 76 | 29 (38.2) | 10.1 | Reference |  | Reference |  |
| 25 to <30 pre-obesity | 92 | 31 (33.7) | 9.0 | 0.88 (0.53-1.46) | 0.62 | 0.81 (0.46-1.44) | 0.48 |
| ≥30 Obesity | 35 | 11 (31.4) | 8.7 | 0.85 (0.42-1.70) | 0.65 | 0.90 (0.37-2.16) | 0.81 |
| ASA | (n=202) |  |  |  |  |  |  |
| I | 59 | 19 (32.2) | 8.4 | Reference |  | Reference |  |
| II | 118 | 41 (34.7) | 9.4 | 1.10 (0.64-1.90) | 0.73 | 1.08 (0.57-2.07) | 0.81 |
| III/IV | 25 | 11 (44.0) | 12.5 | 1.45 (0.69-3.05) | 0.33 | 1.42 (0.58-3.49) | 0.44 |
| T stage | (n=202) |  |  |  |  |  |  |
| T0/T1/T2 | 48 | 6 (12.5) | 2.8 | Reference |  | Reference |  |
| T3 | 118 | 38 (32.2) | 8.4 | 3.01 (1.27-7.11) | 0.012 |  |  |
| T4 | 21 | 12 (57.1) | 22.6 | 7.58 (2.84-20.2) | <0.001 | NE |  |
| No cancer | 15 | 15 (100) | 40.0 | 11.2 (4.28-29.3) | <0.001 | NE |  |
| N stage |  |  |  |  |  |  |  |
| N0 | 108 | 18 (16.7) | 4.0 | Reference |  | Reference |  |
| N1 | 55 | 25 (45.4) | 13.2 | 3.34 (1.82-6.12) | <0.001 | NE |  |
| N2 | 25 | 13 (52.0) | 16.8 | 4.18 (2.05-8.54) | <0.001 | NE |  |
| No cancer | 15 | 15 (100) | 40.0 | 7.96 (3.92-16.1) | <0.001 | NE |  |
| Preoperative radiotherapy | 67 | 22 (32.8) | 8.5 | 0.92 (0.55-1.52) | 0.74 | 1.51 (0.69-3.32) | 0.30 |
| Adjuvant treatment | (n=200)  83 | 37 (44.6) | 12.8 | 1.78 (1.11-2.85) | 0.016 | 1.74 (0.68-4.43) | 0.24 |

^1^Rates=number of events per 100 personyears. ^2^Cox regression stratified on T and N stage, NE=Not estimated.
