# Supplementary material for: Comparison between epidural and intravenous analgesia effects on disease-free survival after colorectal cancer surgery: a randomised multicentre controlled trial
Source: Br J Anaesth. 2021 May 7;127(1):65–74. doi: 10.1016/j.bja.2021.04.002 (PMC8258969; doi:10.1016/j.bja.2021.04.002)
Supplement: Multimedia component 2 [file mmc2.docx]

**Table 3s. Mixed model results of NRS pain intensity at activity between TEA and PCA over time.**

| NRS pain intensity at activity | | | | |  | | | |  | | | |
| --- | --- | --- | --- | --- | --- | --- | --- | --- | --- | --- | --- | --- |
| All subjects | **TEA (n=90)** | | | | **PCA (n=99)** | | | | **TEA vs. PCA** | | | |
|  | **Day1 am**  (n=87) | **Day1 pm**  (n=83) | **Day2 am**  (n=78) | **Day2 pm**  (n=79) | **Day1 am**  (n=96) | **Day1 pm**  (n=93) | **Day2 am**  (n=85) | **Day2 pm**  (n=84) | **Day1 am** | **Day1 pm** | **Day2 am** | **Day2 pm** |
|  | Mean  (SD) | Mean  (SD) | Mean  (SD) | Mean  (SD) | Mean  (SD) | Mean  (SD) | Mean  (SD) | Mean  (SD) | Mean diff  (95 % CI) | Mean diff  (95 % CI) | Mean diff  (95 % CI) | Mean diff  (95 % CI) |
|  | 3.3  (2.2) | 3.3  (2.3) | 3.3  (2.3) | 3.2  (2.1) | 5.1  (2.3) | 4.3  (2.4) | 3.9  (2.3) | 3.6  (2.3) | -1.8  (-2.4 to -1.1)  P<0.001  (P<0.001) | -1.0  (-1.7 to -0.4)  P=0.001  (P=0.001) | -0.6  (-1.3 to 0.1)  P=0.10  (P=0.05) | -0.5  (-1.2 to 0.2)  P=0.16  (P=0.14) |
| Among | **TEA (n=53)** | | | | **PCA (n=55)** | | | | **TEA vs. PCA** | | | |
| OS | **Day1 am**  (n=51) | **Day1 pm**  (n=49) | **Day2 am**  (n=44) | **Day2 pm**  (n=43) | **Day1 am**  (n=53) | **Day1 pm**  (n=50) | **Day2 am**  (n=43) | **Day2 pm**  (n=42) | **Day1 am** | **Day1 pm** | **Day2 am** | **Day2 pm** |
|  | 3.5  (2.6) | 3.3  (2.5) | 3.3  (2.3) | 3.1  (2.0) | 4.8  (2.1) | 3.9  (2.4) | 3.7  (2.3) | 3.3  (2.1) | -1.4  (-2.3 to -0.5)  P=0.002  (P=0.001) | -0.7  (-1.6 to 0.2)  P=0.14  (P=0.045) | -0.5  (-1.5 to 0.4)  P=0.26  (P=0.15) | -0.4  (-1.3 to 0.6)  P=0.45  (P=0.44) |
| Among | **TEA (n=32)** | | | | **PCA (n=34)** | | | | **TEA vs. PCA** | | | |
| MIS | **Day1 am**  (n=31) | **Day1 pm**  (n=30) | **Day2 am**  (n=30) | **Day2 pm**  (n=31) | **Day1 am**  (n=33) | **Day1 pm**  (n=33) | **Day2 am**  (n=33) | **Day2 pm**  (n=33) | **Day1 am** | **Day1 pm** | **Day2 am** | **Day2 pm** |
|  | 2.8  (1.5) | 3.1  (2.0) | 3.2  (2.1) | 2.8  (1.8) | 5.4  (2.8) | 4.6  (2.4) | 4.1  (2.6) | 3.7  (2.5) | -2.6  (-3.7 to -1.5)  P<0.001  (P<0.001) | -1.5  (-2.6 to -0.4)  P=0.009  (P=0.009) | -0.8  (-1.9 to 0.3)  P=0.16  (P=0.18) | -0.8  (-2.0 to 0.2)  P=0.13  (P=0.12) |
| Among | **TEA (n=37)** | | | | **PCA (n=44)** | | | | **TEA vs. PCA** | | | |
| MIS and | **Day1 am**  (n=36) | **Day1 pm**  (n=34) | **Day2 am**  (n=34) | **Day2 pm**  (n=36) | **Day1 am**  (n=43) | **Day1 pm**  (n=43) | **Day2 am**  (n=42) | **Day2 pm**  (n=42) | **Day1 am** | **Day1 pm** | **Day2 am** | **Day2 pm** |
| converted | 3.1  (1.6) | 3.2  (2.1) | 3.4  (2.2) | 3.3  (2.2) | 5.4  (2.5) | 4.8  (2.3) | 4.1  (2.4) | 3.9  (2.4) | -2.4  (-3.4 to -1.4)  P<0.001  (P<0.001) | -1.5  (-2.5 to -0.5)  P=0.004  (P=0.004) | -0.6  (-1.6 to 0.4)  P=0.22  (P=0.19) | -0.5  (-1.5 to 0.5)  P=0.29  (P=0.19) |

OS= open surgery, MIS= minimally invasive surgery, SD=Standard deviation, Mean diff=Mean difference, CI=Confidence interval, p values in parenthesis after log transformation on outcome.

**Table 4s. Mixed model results of NRS pain intensity at rest between TEA and PCA over time.**

| NRS Pain intensity at rest | | | | |  | | | |  | | | |
| --- | --- | --- | --- | --- | --- | --- | --- | --- | --- | --- | --- | --- |
| All subjects | **TEA (n=98)** | | | | **PCA (n=104)** | | | | **TEA vs. PCA** | | | |
|  | **Day1 am**  (n=95) | **Day1 pm**  (n=94) | **Day2 am**  (n=89) | **Day2 pm**  (n=89) | **Day1 am**  (n=103) | **Day1 pm**  (n=103) | **Day2 am**  (n=95) | **Day2 pm**  (n=93) | **Day1 am** | **Day1 pm** | **Day2 am** | **Day2 pm** |
|  | Mean  (SD) | Mean  (SD) | Mean  (SD) | Mean  (SD) | Mean  (SD) | Mean  (SD) | Mean  (SD) | Mean  (SD) | Mean diff  (95 % CI) | Mean diff  (95 % CI) | Mean diff  (95 % CI) | Mean diff  (95 % CI) |
|  | 1.0  (1.7) | 1.2  (1.6) | 1.1  (1.7) | 0.9  (1.6) | 2.7  (1.9) | 1.9  (1.6) | 1.6  (1.6) | 1.4  (1.4) | -1.7  (-2.1 to -1.2)  P<0.001  (P<0.001) | -0.8  (-1.2 to -0.3)  P=0.001  (P<0.001) | -0.5  (-1.0 to -0.04)  P=0.034  (P=0.001) | -0.5  (-1.0 to -0.01)  P=0.05  (P=0.003) |
| Among | **TEA (n=59)** | | | | **PCA (n=60)** | | | | **TEA vs. PCA** | | | |
| OS | **Day1 am**  (n=57) | **Day1 pm**  (n=57) | **Day2 am**  (n=52) | **Day2 pm**  (n=51) | **Day1 am**  (n=59) | **Day1 pm**  (n=59) | **Day2 am**  (n=53) | **Day2 pm**  (n=51) | **Day1 am** | **Day1 pm** | **Day2 am** | **Day2 pm** |
|  | 1.3  (2.1) | 1.2  (1.7) | 1.0  (1.8) | 0.8  (1.6) | 2.6  (1.9) | 1.9  (1.7) | 1.7  (1.8) | 1.4  (1.4) | -1.4  (-2.0 to -0.7)  P<0.001  (P<0.001) | -0.7  (-1.3 to -0.3)  P=0.039  (P=0.005) | -0.8  (-1.5 to -0.1)  P=0.018  (P=0.001) | -0.7  (-1.3 to 0.01)  P=0.045  (P=0.002) |
| Among | **TEA (n=34)** | | | | **PCA (n=34)** | | | | **TEA vs. PCA** | | | |
| MIS | **Day1 am**  (n=33) | **Day1 pm**  (n=32) | **Day2 am**  (n=33) | **Day2 pm**  (n=34) | **Day1 am**  (n=34) | **Day1 pm**  (n=34) | **Day2 am**  (n=33) | **Day2 pm**  (n=33) | **Day1 am** | **Day1 pm** | **Day2 am** | **Day2 pm** |
|  | 0.6  (0.8) | 0.9  (1.4) | 1.1  (1.6) | 1.0  (1.5) | 2.8  (2.1) | 1.9  (1.5) | 1.5  (1.2) | 1.4  (1.5) | -2.2  (-2.9 to -1.5)  P<0.001  (P<0.001) | -1.0  (-1.7 to -0.3)  P=0.007  (P=0.002) | -0.4  (-1.1 to 0.3)  P=0.29  (P=0.077) | -0.4  (-1.2 to 0.3)  P=0.22  (P=0.12) |
| Among | **TEA (n=39)** | | | | **PCA (n=44)** | | | | **TEA vs. PCA** | | | |
| MIS and | **Day1 am**  (n=38) | **Day1 pm**  (n=37) | **Day2 am**  (n=37) | **Day2 pm**  (n=38) | **Day1 am**  (n=44) | **Day1 pm**  (n=44) | **Day2 am**  (n=42) | **Day2 pm**  (n=42) | **Day1 am** | **Day1 pm** | **Day2 am** | **Day2 pm** |
| converted | 0.7  (1.1) | 1.1  (1.5) | 1.1  (1.6) | 1.1  (1.5) | 2.8  (2.0) | 2.0  (1.6) | 1.4  (1.2) | 1.4  (1.4) | -2.1  (-2.8 to -1.4)  P<0.001  (P<0.001) | -0.9  (-1.6 to -0.3)  P=0.006  (P=0.002) | -0.2  (-0.9 to 0.5)  P=0.58  (P=0.22) | -0.3  (-0.9 to 0.4)  P=0.43  (P=0.24) |

OS= open surgery, MIS= minimally invasive surgery, SD=Standard deviation, Mean diff=Mean difference, CI=Confidence interval, p values in parenthesis after log transformation on outcome.
